# The neurobiological effects of childhood maltreatment on brain structure, function, and attachment

**DOI:** 10.1007/s00406-024-01779-y

**Published:** 2024-03-11

**Authors:** Akemi Tomoda, Shota Nishitani, Shinichiro Takiguchi, Takashi X. Fujisawa, Toshiro Sugiyama, Martin H. Teicher

**Affiliations:** 1 Research Center for Child Mental Development, University of Fukui, 23-3 Matsuoka-Shimoaizuki, Eiheiji-cho, Yoshida-gun, Fukui 910-1193 Japan; 2 Division of Developmental Higher Brain Functions, United Graduate School of Child Development, Osaka University, Kanazawa University, Hamamatsu University School of Medicine, Chiba University, and University of Fukui, Fukui, Japan; 3 Department of Child and Adolescent Psychological Medicine, University of Fukui Hospital, Fukui, Japan; 4 Developmental Biopsychiatry Research Program, McLean Hospital, Belmont, USA; 5 Department of Psychiatry, Harvard Medical School, Boston, USA

**Keywords:** Childhood maltreatment (child abuse and neglect), DNA methylation, Neuroimaging, Oxytocin, Reactive attachment disorder

## Abstract

Childhood maltreatment is a risk factor for psychopathologies, and influences brain development at specific periods, particularly during early childhood and adolescence. This narrative review addresses phenotypic alterations in sensory systems associated with specific types of childhood maltreatment exposure, periods of vulnerability to the neurobiological effects of maltreatment, and the relationships between childhood maltreatment and brain structure, function, connectivity, and network architecture; psychopathology; and resilience. It also addresses neurobiological alterations associated with maternal communication and attachment disturbances, and uses laboratory-based measures during infancy and case–control studies to elucidate neurobiological alterations in reactive attachment disorders in children with maltreatment histories. Moreover, we review studies on the acute effects of oxytocin on reactive attachment disorder and maltreatment and methylation of oxytocin regulatory genes. Epigenetic changes may play a critical role in initiating or producing the atypical structural and functional brain alterations associated with childhood maltreatment. However, these changes could be reversed through psychological and pharmacological interventions, and by anticipating or preventing the emergence of brain alterations and subsequent psychopathological risks.

## Introduction

Childhood maltreatment (CM), per the World Health Organization [1], is the abuse and neglect of children aged < 18 years. It includes all types of physical, sexual, and psychological/emotional violence, and the neglect of infants, children, and adolescents by parents, caregivers, and other authority figures, at home and in settings such as schools and orphanages. CM markedly increases the risks for adverse psychiatric outcomes. A recent comprehensive meta-analysis showed that CM is a cross-diagnostic risk factor for a variety of psychiatric diagnoses [2]; CM is a risk factor for (1) psychiatric diagnoses such as anxiety, depression, psychosis, complex post-traumatic stress disorder (PTSD), substance use, and (2) syndromes, symptoms, and personality traits such as sleep disruption, antisocial behavior, suicide and non-suicidal self-injury, and it impairs cognitive abilities [3–9].

CM is a potent risk factor for atypical brain development with marked effects on morphology, function, and circuitry [10–14]. It exerts a more significant impact on trajectories of brain development than categorical psychiatric disorders [15–17]. The nature and intensity of the effects greatly depend on the type and timing of CM during sensitive developmental periods. Moreover, CM appears to be a critically important etiological factor as maltreated and non-maltreated individuals with the same primary psychiatric diagnosis appear to be clinically and neurobiologically distinct, with the maltreated ecophenotype showing an earlier onset, more comorbidities, a more pernicious clinical course, and an array of alterations in the structure, function, and connectivity of stress-susceptible brain regions [18].

Hundreds of studies have reported associations between CM and alterations in brain structure, function, connectivity, and network architecture in children or adults. A recent meta-analysis focused on gray matter (GM) abnormalities linked to CM, incorporating 30 Voxel-Based Morphometry (VBM) studies with 1264 individuals having a history of CM and 1201 non-maltreated controls [19]. The meta-analysis revealed distinctive GM changes in young individuals exposed to maltreatment, including reduced GM in the cerebellum and increased GM in the bilateral middle cingulate/paracingulate gyri and bilateral visual cortex when compared to maltreated adults. Notably, opposing GM changes were observed in the bilateral middle cingulate/paracingulate gyri between maltreated adults (decreased) and maltreated children/adolescents (increased). These differences suggest that the impact of CM on GM varies based on the proximity to maltreatment events, potentially influencing the developmental trajectory of brain structure. This vast field cannot be covered in a single review article.

Maturation of the human brain is regionally specific, non-linear, and heterogenous between individuals [20–22]. Pediatric brain maturation is characterized by increased connectivity among disparate brain regions, environmentally driven specialization of brain circuitry, and a changing balance between earlier maturing limbic regions and later maturing prefrontal regions that continues to undergo substantial changes well into the third decade [21, 23]. Brain regions and fiber tracts have age-dependent developmental windows and may have sensitive periods when they are maximally susceptible to environmental factors [24–27]. Overall, trajectories of brain development are likely affected by CM during periods of rapid developmental changes, which predominantly occur during early postnatal development and adolescence [25, 28]. Healthy brain development requires exposure to an array of expected species-specific stimuli and protection from threats or stressors that may overwhelm the developing brain [29]. During childhood, caregivers play a pivotal role in providing the necessary stimulation and protection against the effects of stress; this role is best exercised through a secure attachment. Hence, early neglect, deprivation, and threats to the attachment bond may have particularly deleterious effects on brain development [30]. While early childhood is characterized by the overproduction of axons, dendrites, synapses, and receptors, adolescence is characterized by rapid pruning [31, 32]. In addition, adolescence is a peak period of vulnerability to the emergence of psychiatric disorders [33, 34] and susceptibility to maltreatment and peer bullying [35].

Sensory systems develop early as myelination of sensory pathways begins in utero and takes place at a rapid rate during the early infancy. Further, the development of sensory systems is experience dependent and highly plastic during childhood [36]. Hence, sensory systems are not only vulnerable to the effects of CM but may be affected in important ways by the quality of a child’s attachment to their primary caregivers. Indeed, attachment theory suggests that the quality of early relationships and attachment bonds with caregivers can significantly impact the development of sensory processing abilities [37]. Children who have experienced maltreatment, such as neglect, abuse, or inconsistent caregiving, may develop insecure or disorganized attachment styles [38] that can disrupt the normal development of sensory systems, leading to problems in perceiving, processing, and integrating sensory information. For example, children with insecure attachment patterns may exhibit heightened sensitivity or hypervigilance to sensory stimuli, resulting in sensory over-responsivity. They may struggle regulating their emotional responses to sensory input or filtering and organizing sensory information. In contrast, children with disorganized attachment patterns may display inconsistent or unpredictable responses to sensory input, causing challenges in effectively integrating sensory information and forming coherent perceptual experiences [38–40].

These sensory system problems can manifest in various ways, such as sensory seeking or avoidance behaviors, sensory sensitivities or aversions, poor modulation of sensory input, and difficulties with attention, self-regulation, and social interactions [37]. Understanding the relationship between CM, disturbed attachment and sensory system problems is crucial for interventions and support services to promote healthy sensory development and address sensory challenges in individuals who have experienced early adversity.

Delineating the effects of early adversity on trajectories of brain development is a complex challenge that researchers have studied in myriad ways. One approach has been to compare adults with different histories of CM to identify associations that may be reflective of the enduring neurobiological consequences of CM in participants who may have experienced the full range of adverse experiences across each year of childhood. Another approach has been to look more specifically at infants or children to identify early neurobiological differences related to CM or disrupted attachment during particular phases of development. Our aim in this review is to highlight some of the key findings that have emerged from these two different approaches.

This narrative review is divided into four chapters. “Diversity of studies on maltreatment-associated neurobiology” delves into the neurobiology associated with maltreatment. “Neurobiology and type and timing of maltreatment” focuses on the atypical consequences of various types of CM on the young adult brain morphology, particularly in the areas regulating sensory systems, which act as our primary filter to the outside world and are modified by experience during sensitive or critical periods [41]. The review emphasizes the significant impact of CM on brain structure, function, connectivity, and network architecture. In “Studies on maltreatment and attachment”, the discussion centers around attachment, including a prospective cohort study on neurobiological correlates of impaired maternal communication and infant attachment and case–control studies on neurobiological alterations in Reactive attachment disorder (RAD), which emerges in some children as a consequence of extreme early social deprivation. Finally, “Effects of CM on genetics and hormones” explores the clinical and neurobiological effects of oxytocin on children, along with studies focusing on the methylation of glucocorticoid-regulated and oxytocinergic genes in maltreated children.

Thus, the central questions that this review answers are: (1) How does CM affect the sensory system and result in phenotypic changes? (2) Which sensitive periods in childhood and adolescence are most vulnerable to the neurobiological effects of abuse? (3) What is the relationship between CM and brain structure, function, connectivity, and network building? (4) How does CM affect psychopathology and resilience? The aim is to comprehensively understand the complex relationships between CM, brain development, psychopathology, and resilience by addressing the abovementioned questions and synthesizing the existing literature. We also conducted a targeted literature review in PubMed on the specific set of findings we bring in as evidence (e.g., CM, sensory systems, neurobiology of attachment, and related terms) to assess if there are more recent findings that either support what we have written or challenge it. This knowledge can contribute to developing effective interventions and support systems for abuse survivors.

## Diversity of studies on maltreatment-associated neurobiology

Initially, imaging studies on the potential neurobiological effects of CM focused on children with verified exposure and psychopathology, notably PTSD [42–44], and adults with psychopathology and retrospectively recalled sexual abuse [45–47]. Subsequently, interest has explosively increased. This includes assessing the potential consequences of exposure to various types of CM [48–54], and influential studies on the neurobiological consequences of severe early neglect, such as the Romanian orphan studies [55–62]. Functional imaging studies have identified prominent effects of CM on systems involved in threat detection and response [63–68], reward processing [68–73], and autobiographical memory [74–76] in children and adults. Structural and functional connectivity studies have tested hypotheses regarding mechanisms responsible for these alterations in threat and reward response [77–81]. Studies have also revealed associations between CM and abnormalities in functional brain networks [82–87] and the structural and functional network architecture of the brain [11, 15, 88, 89]. Multiple studies have explored the link between CM, neurobiology, and risk of psychopathology [77, 90–95], including those assessing ecophenotypic differences between maltreated and non-maltreated individuals with the same psychiatric diagnosis [16, 73, 96–98]. Many individuals reporting exposure to CM are relatively resilient (e.g., have better than expected psychiatric outcomes), and studies have identified brain differences specifically associated with resilience [89, 99–106]. Resilience is not driven by a single biomarker, but by complex processes and influences across multiple levels. Few studies, however, have assessed the effects of treatment on CM-associated brain differences [107–110].

Despite the array of relevant studies, many essential gaps still exist. In most studies, there is a considerable and often uncertain time lapse between exposure to CM and measures of brain structure, function, and connectivity. Hence, little is known about factors mediating these brain changes (e.g., epigenetic modifications, inflammation, trophic factors, stress hormones) and how CM interacts with normal developmental processes [20, 111–113] to affect trajectories of brain development. Mandated reporting requirements are a challenge, prompting important longitudinal investigations, such as the Adolescent Brain and Cognitive Development study, to refrain from systematically collecting information on exposure to the types of early adversity that would need to be reported to child protective services.

Most studies examining the potential neurobiological effects of CM are cohort studies in which comparisons are made between exposed and unexposed cohorts to identify differences in brain measures and clinical outcomes. The alternative observational strategy is the case–control study in which participants are selected based on the presence or absence of specific clinical criteria (e.g., depression, PTSD) [114]. These groups can be compared for differences in exposure rates to CM and other factors. A vital advantage of the case–control study is that it can be effectively used to study risk factors for rare disorders, such as RAD.

## Neurobiology and type and timing of maltreatment

CM alters brain structures, impacting the hippocampus, anterior cingulate, ventromedial and dorsomedial cortices, and fiber tracts. Teicher et al. reviewed consistent findings of altered amygdala response, reduced ventral striatal response, disrupted prefrontal–amygdala connectivity, and increased precuneus volume in maltreated individuals [115]. Despite similar psychiatric diagnoses, maltreated and non-maltreated individuals exhibit distinct clinical, neurobiological, and genetic differences. The impact of maltreatment on resilient individuals, without psychopathological symptoms, suggests underlying compensatory mechanisms for stress-related neurobiological changes.

Ohashi et al. used diffusion tensor imaging (DTI) MRI and tractography on 262 unmedicated, healthy 18–25-year-old individuals. Those with moderate-to-high maltreatment exposure (*n* = 140) displayed lower global network metrics, including degree, strength, and efficiency, indicating potential links to psychopathology. Major depression, anxiety, and ADHD (attention-deficit hyperactivity disorder) history did not independently influence global network measures, emphasizing the pivotal role of maltreatment in understanding network differences and psychopathology [15]. Another study examined corpus callosum (CC) abnormalities in psychiatric disorders and their association with maltreatment [116]. In 345 healthy 18–25-year-olds, AI predictive analytics identified maltreatment types and timings. Males showed CC alterations in axial diffusivity linked to emotional abuse or neglect, while females had alterations in radial diffusivity and fractional anisotropy associated with early physical neglect. Sex differences in CC were limited to the genu, emphasizing the need to consider maltreatment and sex in understanding the role of CC in psychiatric disorders.

Further, abnormal amygdala responses to threat-related stimuli are linked to various disorders. CM, a significant risk factor, correlates with atypical amygdala function. Zhu et al. investigated impact of maltreatment timing on amygdala response, finding prepubertal differences in associations. Understanding such nuances aids targeted interventions for at-risk youth [64]. Thus, neurobiological distinctions between maltreated and non-maltreated individuals underscore the need for DSM-5 updates, proposing a Developmental Trauma Disorder and modified diagnostic categories to enhance precision and treatment outcomes [117].

**Fig. 1:**
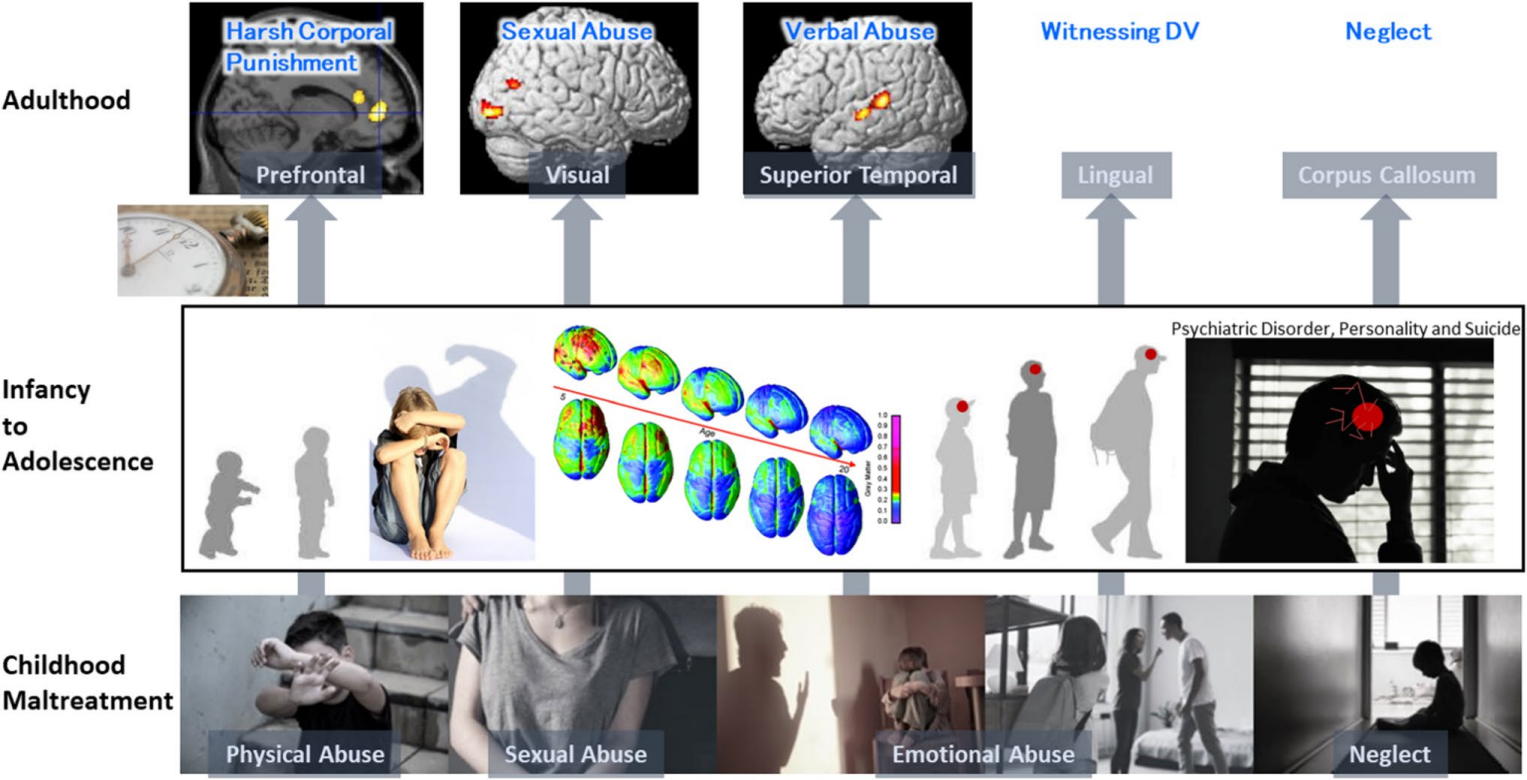
Structural changes depending on the type of childhood maltreatment (CM) to see what brain structures are affected by exposure to CM and if the type of maltreatment matters [51–53, 118]. (Top from the left) Prefrontal cortex gray matter volume (GMV) reduction in participants who experienced harsh corporal punishment [118], visual cortex GMV reduction in participants who experienced repeated exposure to childhood sexual abuse [118], superior temporal gyrus GMV increase in participants who experienced parental verbal abuse [52], lingual gyrus GMV reduction in participants who witnessed interparental violence during childhood [53], and corpus callosum volume reduction in participants who experienced neglect [236]. (Middle) Right lateral and superior views of the dynamic sequence of GM maturation over the cortical surface. Copyright (2004) National Academy of Sciences, USA [23]. (Bottom from the left) Illustrations of physical abuse, sexual abuse, emotional abuse, and neglect

Previously, Tomoda and Teicher et al. investigated structural brain abnormalities with volumetric magnetic resonance imaging (MRI) in young adults with CM identifying CM-specific structural changes supporting the hypothesis that the nature of maltreatment matters [48–53, 118] (Fig. 1). Screenings were conducted on 1455 volunteers to select specific subsets of participants with moderate-to-severe exposure to one particular type of CM and minimal exposure to other types of CM or adversity for T1-weighted and diffusion tensor MRI.

### Childhood sexual abuse (CSA)

First, Tomoda et al. investigated brain structures in young adults with repeated exposure to CSA [118] unaccompanied by exposure to physical abuse, neglect, or witnessing domestic violence (WDV). Seventy-five percent of CSA victims experienced CSA by individuals outside their families. Participants with repeated exposure to CSA (*n* = 23, 20.2 ± 1.3 years) had a 12.6 and 18.1% GMV reduction in their right and left primary visual and visual association cortices, respectively, compared with healthy control participants (*n* = 14, 19.0 ± 1.1 years) [118]. This reduction was directly related to the duration of sexual abuse before 12 years of age. Moreover, cortical surface-based analysis indicated that the GMV of CSA participants was reduced in the left fusiform (face recognition), left middle occipital, and right lingual gyri (global aspects of figure recognition) regions. Heim et al. [119] reported thinning of the specific portion of the somatosensory cortex that is critical to the perception of touch in the clitoris and surrounding genital area of women who had experienced penetrative sexual abuse (*n* = 51, 27.0 ± 7.0 years). Bremner et al. [120] reported decreased blood flow in the visual association cortex of women with CSA and PTSD (*n* = 10 with PTSD, 35.0 ± 6.0 years;* n* = 12 without PTSD, 32.0 ± 8.0 years). Berman et al. [121] reported that GMV in the lingual gyrus and intracalcarine cortex of women who had experienced sexual assault was inversely correlated with their degree of assault-related self-blame (*n* = 68, 19–37 years).

Hubel and Wiesel [122] identified environmentally sensitive periods of brain development, particularly in the organization of the visual cortex. Tomoda et al. tested the hypothesis that there are regionally specific developmentally sensitive periods when brain regions are maximally affected by exposure to CM. In association with CSA, Tomoda et al. found reductions in hippocampal volume at ages 3–5 and 11–13 years, corpus callosum volume at ages 9–10 years, and frontal cortex volume at ages 14–16 years [26]. Tomoda et al. also reported that the degree of reduction in visual cortex GMV was directly related to the duration of sexual abuse before 12 years of age [118], which is consistent with Hubel and Wiesel’s observation that the mammalian visual cortex is highly plastic until puberty [122]. Subsequent studies have also provided evidence for regionally specific age-dependent windows of vulnerability to CM [10, 63, 64, 116, 123–126].

### Parental verbal abuse (PVA)

Similarly, Tomoda et al. assessed regionally specific effects of PVA among individuals with moderate-to-severe exposure to PVA and minimal exposure to other forms of adversity from a healthy community sample [52]. Participants with PVA (*n* = 21, 21.2 ± 2.4 years) had a 14.1% GMV increase in the left superior temporal gyrus (STG) auditory cortex compared to healthy controls (*n* = 19, 21.9 ± 2.4 years) [52]. GMV in this cluster was associated most strongly with levels of maternal and paternal verbal aggression and inversely associated with parental education.

Further, diffusion tensor imaging tractography revealed that participants with PVA (*n* = 16, 21.9–37 years) had reduced integrity in three fiber pathways [49]. The most significant difference was in the arcuate fasciculus, which is believed to be a critical substrate for speech, language, and communication. Interestingly, some brain regions respond to stress with a reduction in dendritic arborization (e.g., hippocampus, prefrontal cortex) and others by an increase (e.g., amygdala) [127, 128], which may be related to changes in brain-derived neurotrophic factor levels [129]. Hypertrophy of the STG has been reported in youths with CM and PTSD [130] and in women with comorbid borderline personality disorder (BPD) and PTSD [131]; thus, hypertrophy may be a phenotypic adaptive response to stress in this structure.

### Witnessing domestic violence (WDV)

Tomoda et al. investigated participants who experienced WDV during childhood [53] in a manner similar to that used in the PVA study [52]. Using VBM, Tomoda et al. found that participants (*n* = 22, 21.8 ± 2.4 years) who had visually witnessed multiple episodes of interparental violence during childhood had a 6.1% GMV reduction in the right lingual gyrus (Brodmann area [BA] 18) compared with healthy age-equivalent controls (*n* = 30, 21.6 ± 2.1 years) [53]. Thickness in this region, the secondary visual cortex, and left occipital pole was reduced bilaterally. Regional reductions in GMV and thickness were observed in both susceptible and resilient WDV subjects. WDV also affected the integrity of the left inferior longitudinal fasciculus (ILF) [49], which interconnects the visual and limbic systems and provides emotional and memory responses to what is seen. Olson et al. [132] confirmed that CM was associated with alterations in the integrity of the ILF and was sensitive to exposure to both physical/sexual and emotional forms of abuse (*n* = 32, 20–50 years). Further, Howell et al. [133], in a longitudinal cross-fostering study in primates, provided direct evidence that the ILF was affected explicitly by parental maltreatment. Brain regions involved in that process and conveying the adverse sensory input of the maltreatment may be specifically modified by this experience, particularly in participants exposed to a single type of maltreatment. Exposure to multiple types of maltreatment is more commonly associated with morphological alterations in corticolimbic regions [134]. These findings correlate with preclinical studies showing that the visual cortex is a highly plastic structure [122].

### Harsh corporal punishment (HCP)

Tomoda et al. also investigated the regional effect of HCP during childhood [51]. The criteria for inclusion was spanking of the buttocks by hand with occasional (or more often) use of objects (e.g., strap, hairbrush) beginning before the child’s 12th birthday and lasting for at least 3 years, with a frequency of about 12 episodes or more per year. Participants who experienced HCP (*n* = 23, 21.7 ± 2.2 years) had a 19.1% GMV reduction in the right medial frontal gyrus (medial prefrontal cortex, BA10), 14.5% reduction in the left medial frontal gyrus (dorsolateral prefrontal cortex, BA9), and 16.9% reduction in the right anterior cingulate gyrus (BA24) compared with healthy age-equivalent controls (*n* = 22, 21.7 ± 1.8 years) [51]. The GMV in these identified regions showed significantly positively correlated with performance IQ. Further, HCP was associated with reduced blood flow in dopamine-rich regions, including the caudate, putamen, nucleus accumbens, dorsolateral prefrontal cortex, and substantia nigra [50].

Hence, CM alters the trajectories of brain development to affect sensory systems and circuits involved in threat detection, emotional regulation, and reward anticipation [115, 117, 135], but there is a need to elucidate how CM increases the risk of psychiatric disorders. Several studies have been conducted in the broad area of CM and its neurobiological effects. One suggests that alterations in amygdala volume vary in direction (smaller/larger volumes) and depend on the timing of traumatization [136]. The right amygdala GMV negatively correlated with retrospectively assessed maltreatment severity at 10–11 years of age [10]. Further, the effects on hippocampal volume in individuals who have experienced CM vary by sex during the sensitive period [126].

## Studies on maltreatment and attachment

### Neurobiological consequences of disrupted attachment

An increasing number of studies have assessed the potential impact of alterations in attachment behavior and maternal–infant communication on brain development. These studies include detailed evaluation of attachment behaviors in infancy, followed by longitudinal evaluation of clinical and neurobiological consequences of disorganized attachment. One series of neuroimaging studies was based on a 30-year follow-up of infants assessed for attachment behaviors [137]. The early-life stress cohort consisted of families at or below 200% of the federal poverty line. The quality of mother–infant interaction was directly observed and videotaped in the well-validated Strange Situation Procedure [138] at 18 months of age and reliably coded using the Atypical Maternal Behavior Instrument for Assessment and Classification [139]. Disorganized attachment and impaired maternal communication at 18 months were associated with enlarged left amygdala volume at around 30 years of age [140]. Contrastingly, right amygdala volume negatively correlated with retrospectively assessed maltreatment severity at 10–11 years of age [10]. The amygdala plays a critical role in threat detection and response, leading to the hypothesis that the left amygdala may be specialized for detecting threats to the attachment bond or abandonment. Conversely, the right amygdala may be specialized for detecting physical, sexual, or emotional threats [140]. This is appealing as the left hemisphere is generally more involved in motivating approach behaviors, which may be most helpful when the attachment bond is threatened, while the right hemisphere is more involved in motivating avoidance behaviors [141]. Khoury et al. [142] reported left hippocampal volume in adulthood was associated with maternal withdrawal during infancy, but not with other components of disrupted parenting. Left hippocampal volume was also linked to increased BPD features and tendencies towards suicidality and self-injury. In addition, left hippocampal volume partially mediated the connection between early maternal withdrawal and later suicidality/self-injury [142].

More recently, Lyons-Ruth et al. [143] assessed attachment behaviors in 57 mother–infant dyads—the infants underwent MRI during natural sleep at a mean age of 12.3 months. The aim was to explore the neural correlates of maternal withdrawal and negative/inappropriate interactions with infants during infancy, as these behaviors may represent different aspects of early deprivation and threat, respectively. Maternal withdrawal and negative/inappropriate behaviors were observed and coded from the Still-Face Paradigm when infants were four months old. Maternal withdrawal was associated with lower infant GMV, while negative/inappropriate interaction was linked to lower overall WMV. These effects were not influenced by age. Maternal withdrawal was also connected to reduced right hippocampal volume at older ages. Exploratory analyses of white matter tracts revealed that negative/inappropriate maternal behavior specifically correlated with reduced volume in the ventral language network.

### Reactive attachment disorder (RAD)

The previous study explored the potential neurobiological consequences of impaired or withdrawn maternal behaviors leading to alterations within the typical constellation of attachment behaviors (i.e., secure, insecure, disorganized). However, more severe neglect/deprivation can cause profound alterations in attachment-related behaviors, as manifested in Reactive attachment disorder (RAD). RAD is characterized by persistent difficulties in social and emotional relationships and emotionally withdrawn/inhibited behaviors toward caregivers. The belief that CM (specifically social–emotional neglect) is of critical importance in the genesis of this disorder is apparent as a DSM-5 diagnosis requires either social neglect or deprivation in the form of a persistent lack of having the basic emotional needs for comfort, stimulation, and affection met by caregiving adults or rearing in unusual circumstances that severely limit opportunities to form selective attachments [144]. Children with RAD display difficulties with cognition and behavior, and they manifest social and emotional disturbances [145, 146]. They will likely experience disturbed and developmentally inappropriate social interactions throughout adulthood [147, 148]. The prevalence of RAD is approximately 1% in the general population [149] and 19–40% in children in foster care [150, 151]. Hence, case–control studies play an important role in delineating the underlying neurobiological alterations associated with profound alterations in attachment behaviors. Our laboratory is one of the few research institutions that have investigated RAD using brain imaging [123, 152–154]. Due to severe maltreatment, the reward and sensory system pathways mediating emotion regulation are altered in children with RAD compared to those in healthy controls [123, 125, 152–154]. In RAD, the brain appears less likely to experience a sense of accomplishment. RAD might be associated with altered brain function and pathways affecting emotion regulation, leading to core RAD symptoms such as the absence of focused attachment behavior toward a preferred caregiver and emotion regulation disturbances [155, 156]. Contrarily, Leblanc et al. [157] examined attachment stability to the mother in 15-month-old infants and subsequently measured their regional brain GMV at 10 and 11 years of age. More stable attachments were associated with more significant GMV in the superior temporal sulcus, temporal–parietal junction, and precentral gyrus. Thus, attachment stability may influence the development of brain regions involved in social cognition and emotion.

### Neurobiology of attachment and role of oxytocin (OXT)

Attachment is the deep emotional bond that forms between an infant and their primary caregiver, typically the mother [37]. It is a fundamental aspect of human development that is essential in shaping an individual’s relationships and overall well-being. The quality of attachment bonds formed during infancy and childhood has long-term effects on a person’s social, emotional, and cognitive development. Attachment relationships formed during this period serve as models for future relationships and influence a person’s ability to form and maintain healthy social connections throughout life. Attachment bonds are believed to be established through infant–caregiver interactions that are characterized by responsiveness, sensitivity, and consistent care. A secure attachment is formed when the caregiver is consistently available and provides a responsive, safe, nurturing environment. This secure foundation allows the infant to explore their surroundings, knowing that they can seek comfort and support from the caregiver as needed.

The neuropeptide OXT has been implicated in mediating numerous prosocial effects ranging from approach/avoidance behavior [158] to interpersonal trust [159]. Oxytocinergic neurons project to brain regions involved in social and maternal behavior [30]. Animal and human studies showed that OXT is critical for mother–infant bonding and pair-bonding (i.e., secure attachment formation with a primary caregiver) [160, 161]. Bonds are based on OXT and dopamine action within the nucleus accumbens that promote the synaptic plasticity required for bonding with the infant. Attachment between a parent and their infant induces the release of OXT, which may mediate anti-stress effects [162–164]. Notably, early-life experiences appear to have a long-lasting effect on the OXT system.

A paucity of research concerns paternal nurturing behavior. For a considerable time, parenting has primarily been a maternal responsibility [37]. Therefore, researchers and academia have devoted more attention and resources to studying the parenting behavior of mothers [165, 166]. Research on the parenting behavior of fathers is relatively recent and has been influenced by traditional gender role biases. Further, social expectations and role images of fathers have constrained research on paternal behavior. Traditionally, fathers have been viewed as providers of financial support, while mothers are expected to provide emotional care and parenting [167]. Therefore, there has been a decreased interest in research on paternal child-rearing behaviors. Moreover, research on the parenting behaviors of fathers involves accessibility constraints; in some cultures and social contexts, the involvement of the father may be more limited than that of the mother. Work environments or institutional issues may also limit the ability of fathers to devote time to child-rearing. These challenges may prevent researchers from obtaining sufficient data and participants.

Contrastingly, CM dysregulates the brain’s oxytocinergic system, causing anxiety and dysfunctional attachment patterns [168, 169]. Evidence for long-term consequences of negative child–caregiver relationships include differences in OXT that are centrally involved in social relationships. For example, lower-than-average levels of OXT are found in the cerebrospinal fluid (CSF) of adult women with a history of CM [170] and in the urine of socially deprived children interacting with their mothers [171]. Furthermore, there is an association between a history of CM and abnormal OXT levels in the saliva and blood, specifically in adults: increased salivary OXT levels are seen even with a history of less severe forms of CM [172] and lower OXT receptor (*OXTR*) protein expression is found in peripheral blood mononuclear cells [168]. Children exposed to CM show lowered salivary OXT levels with visual attention to social cues [173], presumably hyper-regulated diurnal OXT secretion to cope with the environment, survive, and thrive [174]. There is a relationship between maladaptive mother–child interaction and OXT secretion, as evidenced by reports of decreased blood OXT levels in mothers with BPD who experience disrupted interaction with their child [175].

Further, harsh parenting experiences moderate the OXT effect on the use of excessive force while listening to infant crying sounds [176]. Thus, the current concepts of endogenous OXT effects emphasize the vital role of neuropeptide OXT in regulating mother–infant bonding and attachment in children subjected to maltreatment and lacking attachment or bonding with a primary caregiver [169, 173, 174, 177–179].

The proportion of children exposed to severe CM who can recover from adversity and demonstrate high competency levels across multiple domains (e.g., behavioral, emotional, and educational) is 10–25% [180]. The exogenous intranasal administration of OXT also alters attachment representations later in life, with less anxiously attached individuals remembering their mother as more caring after intranasal OXT administration [160]. Conversely, more anxiously attached individuals remember their mother as less caring [181]. Thus, early intervention and treatment combined with supplementary OXT administration may be one approach to foster the formation of a stable attachment in maltreated children with impaired attachments [182]. In a preliminary placebo-controlled study, intranasal OXT enhanced the activity of the bilateral caudate in children and adolescents with RAD, albeit at a relatively lenient statistical threshold [154]. Although OXT is ineffective for treating autism spectrum disorder symptoms [183], given its central role in attachment formation and prosociality, it may strengthen the neural basis of the reward system for attachment change in individuals with RAD. Further investigations are needed to elucidate the molecular and neural mechanisms of intranasal OXT and identify novel targets for maltreated children with trauma-related disorders such as RAD.

## Effects of CM on genetics and hormones

Exposure to CM profoundly affects the developing brain [28, 135]. Neurological changes due to unfortunate early-life stress can cause lifelong psychiatric sequelae [184]. Hence, there is a great interest in understanding how CM gets “under the skin” to affect brain development. A fundamental mechanism may include early-life stress-induced epigenetic modifications. The human epigenome changes throughout life, and of particular relevance to brain development and risk for psychopathology are epigenetic modifications to genes involved in glucocorticoid regulation, leading to a blunted or enhanced hypothalamic–pituitary–adrenal (HPA) axis response to stress [185–188]. Frequent and chronic exposure of the developing brain to excessive glucocorticoids can affect gene expression, myelination, neural morphology, neurogenesis, synaptogenesis [28, 189, 190], and immune and inflammatory processes (for review, see Andersen [191]), leading to alterations in trajectories of brain development. The HPA axis is exceptionally responsive to stress during adolescence [192, 193]. Shirtcliff et al. introduced a novel hypothesis that specific stressors can produce different developmental effects during adolescence when the HPA and hypothalamic–pituitary–gonadal axes are jointly regulated (for review, see Shirtcliff et al. [194]). Sex hormones exert important organizing effects on brain development during the peripubertal period and work with stress hormones to foster biological and social adaption to stressful and novel events.

### Epigenetic modification to OXT-related genes

**Fig. 2:**
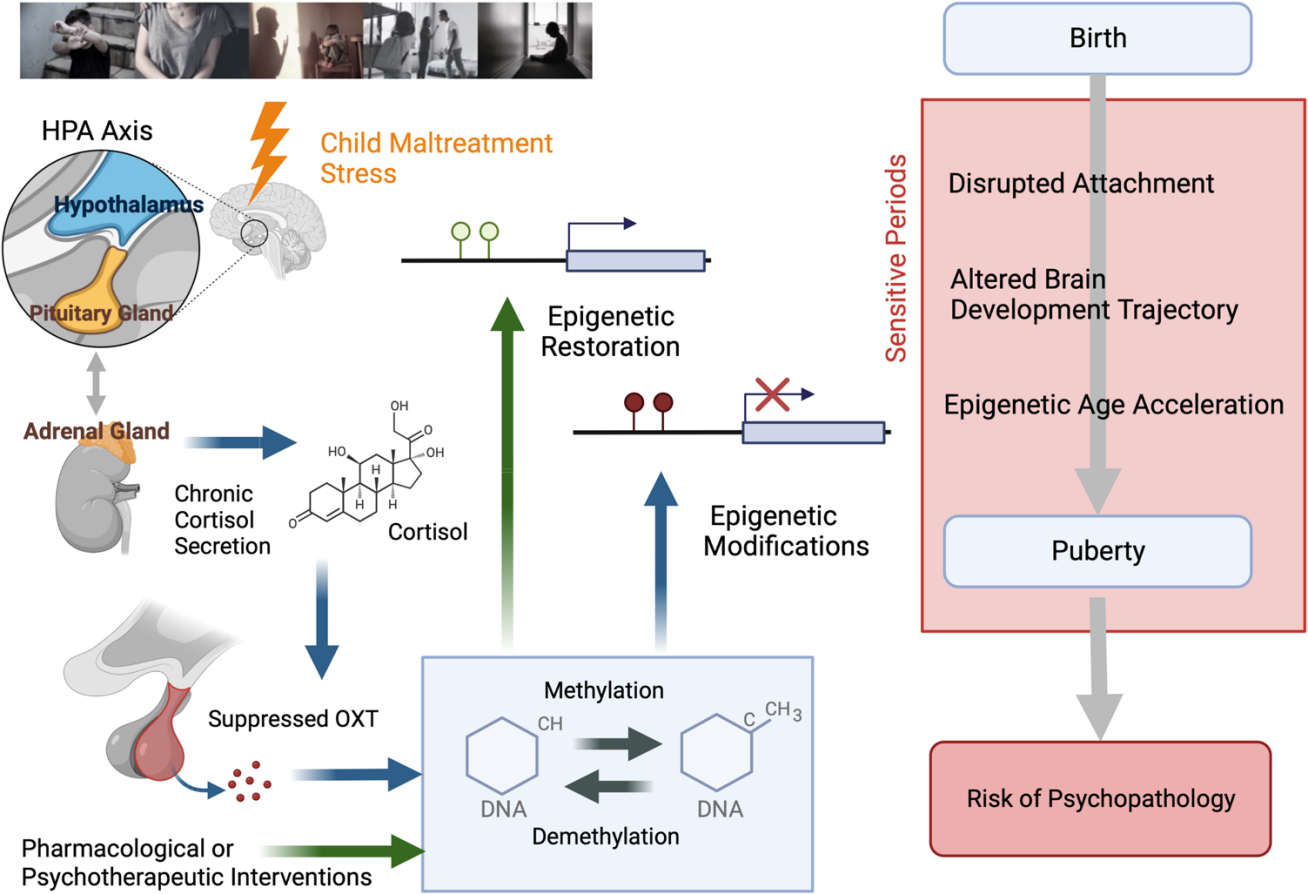
An overview of the possible role of epigenetic modification due to childhood maltreatment (CM). The long-term effects of CM on hypothalamic–pituitary–adrenal axis function, including dysregulation of cortisol secretion, are presumed to result in suppressed oxytocin (OXT) secretion and altered methylation of OXT-related genes and other genes. Thus, exposure to CM during sensitive periods may lead to altered brain developmental trajectories and an increased risk of psychopathology after childhood and adolescence. Epigenetic modifications, particularly DNA methylation is considered critical events in this process and can be targeted by pharmacological or psychotherapeutic interventions. Part of the figure was designed using resources from Biorender.com and http://www.istockphoto.com

Glucocorticoids regulate OXT synthesis and secretion in response to chronic stress [195] (Fig. 2). The possibility of epigenetic regulation of *OXTR* gene methylation and attachment formation has recently been assessed in several studies (for review, see Darling Rasmussen and Storebø [196]); however, conclusive evidence in humans is lacking. *OXTR* gene methylation density was associated with perinatal depression, attachment style, and trauma history, but attachment insecurity was not a significant mediator [197]. Per Ein-Dor et al. [198], attachment avoidance in young adults was specifically associated with increased OXTR and NR3C1 promoter methylation. We investigated how CM alters *OXTR* gene promoter methylation in children who have experienced CM compared with TD children (CM: *n* = 44 vs. TD: *n* = 41, 6–20 years). A specific region of the *OXTR* gene (CpG 5,6) was highly methylated in maltreated children and a neuroimaged subset (*n* = 24, 31) was associated with reduced orbitofrontal cortex GMV [199]. This makes sense, given that OXTR localizes in reward-associated circuits.

Although most oxytocinergic system studies focus on the receptor gene, the involvement of the *OXT* gene that synthesizes the OXT peptide itself cannot be ignored. Haas et al. [200] reported that individuals with lower levels of OXT methylation (presumably linked to higher OXT expression) displayed more secure attachments, an improved ability to recognize emotional facial expressions, and greater superior temporal sulcus activity during social–cognitive functional MRI (fMRI) tasks than participants with higher OXT DNA methylation. Consistently, we found that the *OXT* gene promoter region was more widely methylated in children with CM than in TD controls. In addition, whole-brain VBM analysis showed that higher methylation was associated with reduced left superior parietal lobe GMV. This region is critically involved in visual spatial attention control [201], which may provide a neurobiological mechanism for our finding that maltreated children had deficient gaze fixation for the eye area of the human face and reduced salivary OXT [173]. We also found that *OXT* methylation was associated with reduced right putamen activation during a monetary reward task. The putamen is part of the reward system, and reduced reward system activation is one of the most consistently observed fMRI abnormalities in individuals with CM. The finding that the reward system response is greatly regulated by the degree of *OXT* gene methylation supports the hypothesis that maltreatment affects brain development through epigenetic alterations. Moreover, promoters of the *OXT* and *OXTR* genes may play a significant role in this process.

## Conclusions and future directions

This narrative review discusses phenotypic alterations in sensory systems related to specific types of CM, vulnerable periods to its neurobiological effects, and the connections between maltreatment and brain structure, function, connectivity, and network architecture. It also covers psychopathology and resilience in this context. Future studies should embrace a complexity theory approach, investigating various biological levels and their temporal dynamics, which requires longitudinal studies on neurobiological mechanisms considering genetics, endocrine and immune systems, brain structure and function, cognition, and environmental factors. Embracing complexity can foster collaboration and guide efforts to promote resilience in those affected by CM (for review, see study by Ioannidis et al. [202]).

The review delves into neurobiological changes linked to disturbances in maternal communication and attachment, using laboratory measures and case–control studies to examine such alterations in children with maltreatment histories, including reactive attachment disorders. In addition, the acute effects of oxytocin on reactive attachment disorder, maltreatment, and the methylation of oxytocin regulatory genes are discussed. The passage highlights the potential role of epigenetic changes in initiating structural and functional brain alterations due to CM, suggesting the possibility of reversal through psychological and pharmacological interventions.

Understanding these variations is crucial for tailoring therapeutic interventions and support systems. Clinically, the identified brain structural and functional changes associated with CM underscore the need for a comprehensive approach to mental health care [18]. Healthcare professionals should consider the potential neurological impact of CM when assessing and designing treatment plans for individuals with such histories. Early detection of these brain alterations may inform targeted interventions aimed at mitigating the long-term psychological consequences of CM, promoting more effective therapeutic strategies, and improving overall clinical outcomes. Meanwhile, trauma-focused interventions such as Cognitive Behavior Therapy (CBT) trauma-focused strategies and Eye Movement Desensitization and Reprocessing (EMDR) and preventive measures should be implemented in clinical care to reduce CM and, ultimately, the prevalence of mental illness [203].

Further, we need to establish longitudinal assessments after CM to develop more effective diagnostic and treatment strategies, such as those based on regional brain structural and functional differences and epigenetic targets. Specifically, if the inhibition of OXT synthesis caused by promoter methylation results in adverse symptoms, early intervention and treatment combined with interventional supplementation of oxytocinergic function through intranasal OXT administration may be effective for improving clinical outcomes [154, 182, 204]. As most mental disorders result from deviations from the path of typical development, with different illnesses deviating at other times or in different ways (i.e., the neurodevelopmental hypothesis) [205, 206], a greater understanding of these specifics is the key to more effective preventative and interventional measures to ameliorate their effects. The comprehensive goal of elucidating the mechanisms and influences on brain development during adolescence is to improve the lives of children and their families. It is also crucial for optimizing healthy development [207]. To this end, we aim to conceive a synergy of genomic, neurobiological, psychosocial, and developmental components with technical advances in artificial intelligence and tele-present robotic care. Although ambitious, a strategy of this scope is needed to address these fundamental questions about the influence of early adversity on brain development and mental health, and to improve outcomes [117, 208–210].

This review has some limitations. First, there are several shortcomings, particularly “confirmation biases,” inherent to narrative reviews being less methodologically rigorous than are systematic reviews [211]. These biases can reduce the reliability and scientific validity of narrative reviews.

Second, the main limitation includes the small sample sizes and modest number of replication studies. However, while it may take thousands of participants to reliably identify brain structures associated with complex cognitive or mental health phenotypes [212], translational studies have shown that early experience exerts a prepotent effect on brain development, which can be reliably observed with small sample sizes [122, 213–216]. Moreover, the study designs employed in the works presented in the first part of this review—which focus on individuals who have suffered one type of CM (exceptions) and exclude those who have suffered multiple types (typical cases)—pose another risk of bias.

Third, it is challenging to delineate the neurobiological effects of exposure to specific types of CM and identify sensitive exposure periods, as many individuals were exposed to multiple types of maltreatment over many years. We have addressed this problem through selective recruitment [51–53, 118] and machine learning strategies to identify the most important predictor variables in highly collinear data sets [63, 64, 116, 123, 125, 126]. Nevertheless, there is a need to replicate studies using large sample sizes and prospective longitudinal designs.

Fourth, DNA samples such as blood, buccal mucosa, and saliva may not reflect brain conditions due to tissue specificity of methylation patterns. However, correlations between peripheral and brain methylation levels have recently been confirmed in independent databases constructed with Caucasian (https://han-lab.org/methylation/default/imageCpG) [217] and Japanese samples (https://snishit-amaze-cpg.web.app) [218]. Fifth, this review has focused on a few target genes, but a data-driven exploration at the genome-wide level is needed.

Lastly, the results of previous studies should be interpreted carefully, as prospective and retrospective evaluations of CM differ in identified population groups [219]. Among the 101 studies, 13 were prospective and 88 retrospective evaluation studies. In total, 38 used the Childhood Trauma Questionnaire (CTQ), 14 used the Maltreatment and Abuse Chronology of Exposure Scale (MACE), 13 used the Adverse Childhood Experiences Questionnaire (ACEs), and 5 used the Early Trauma Inventory [220] as self-report measures.

CM experiences can be assessed using various methods, including retrospective self-report measures and interviews. Self-report measures typically involve participants completing questionnaires that assess their history of maltreatment, such as the CTQ [221], MACE scale [222], or ACEs questionnaire [223]. These measures rely on participant recollection and the perception of their experiences. Interview-based assessments involve structured or semi-structured interviews conducted by trained professionals to obtain accurate information. These measures can include structured interviews, such as the Childhood Trauma Interview (CTI) [224] or Childhood Experience of Care and Abuse (CECA) interview [225], which involve detailed questioning about various forms of maltreatment. Ideally—using a combination of interview-based, self-report, and caregiver-report measures—a validated instrument designed to assess CM should be used to obtain accurate information. Further, earlier research concerning surveys conducted among children with legally verified CM, including children with CM verified by the Department of Social Services, is outdated, and this approach has as many disadvantages as advantages. These considerations suggest careful interpretation of the studies presented in the current review. Addressing the issue of heterogeneity in definitions of CM and data collection methods requires a comprehensive and standardized approach. By taking a multi-faceted and collaborative approach (1) establish clear definitions, (2) standardize assessment tools, (3) provide training and guidelines, (4) cross-disciplinary collaboration, and (5) longitudinal studies, it would be possible to mitigate the challenges associated with heterogeneity in definitions of CM and data collection methods, ultimately improving the comparability and reliability of research findings in this critical area.

Although methylation is considered the most stable form of epigenetic modification, there is evidence that DNA methylation is plastic from childhood to adulthood. Therapeutic interventions may alter methylation patterns and reduce the biological risks posed by CM [226–228]. Moreover, psychological and pharmacological interventions may reverse these epigenetic changes and prevent or delay the development of childhood-onset psychiatric disorders and psychopathology in adulthood caused by CM exposure [229].

Recent studies have focused on the role of CM in adolescent-onset psychiatric disorders [59, 230–233]. Exposure to stress during periods of rapid brain change, particularly during adolescence, may make youth exceptionally susceptible to developing mental illness [25]. Many cross-sectional and longitudinal cohort studies report potential sensitive period effects for psychopathology; however, for most studied outcomes, no consistent period of increased susceptibility has been identified (see review by Schaefer et al. [234]). One potential explanation is that studies reporting later ages of peek susceptibility [35, 64] include peer physical and emotional bullying as risk factors, whereas studies on earlier sensitive periods do not. Findings of distinct stages of vulnerability for regional brain development support the sensitive period hypothesis [235]. Although firm conclusions cannot be drawn now, identifying the most important type/time risk factors for various psychiatric disorders will be immensely valuable in developing strategies for prevention, preemption, and treatment. Further, we anticipate that sensitive exposure periods for developing specific psychiatric disorders will intersect with windows of vulnerability for brain systems critically involved in the genesis of these disorders and will shed new light on gene–development–environment interactions that lie at the heart of most psychiatric vulnerabilities [14].
